# Cerato-platanins: a fungal protein family with intriguing properties and application potential

**DOI:** 10.1007/s00253-014-5690-y

**Published:** 2014-04-01

**Authors:** Romana Gaderer, Klaus Bonazza, Verena Seidl-Seiboth

**Affiliations:** 1 Research Area Biotechnology and Microbiology, Institute of Chemical Engineering, Vienna University of Technology, Gumpendorfer Strasse 1a, 1060 Vienna, Austria; 2 Institute of Materials Chemistry, Vienna University of Technology, Getreidemarkt 9, 1060 Vienna, Austria

**Keywords:** Cerato-platanin, Chitin, Self-assembly, Protein layer, Hydrophobin, Expansin

## Abstract

Cerato-platanin proteins are small, secreted proteins with four conserved cysteines that are abundantly produced by filamentous fungi with all types of lifestyles. These proteins appear to be readily recognized by other organisms and are therefore important factors in interactions of fungi with other organisms, e.g. by stimulating the induction of defence responses in plants. However, it is not known yet whether the main function of cerato-platanin proteins is associated with these fungal interactions or rather a role in fungal growth and development. Cerato-platanin proteins seem to unify several biochemical properties that are not found in this combination in other proteins. On one hand, cerato-platanins are carbohydrate-binding proteins and are able to bind to chitin and *N*-acetylglucosamine oligosaccharides; on the other hand, they are able to self-assemble at hydrophobic/hydrophilic interfaces and form protein layers, e.g. on the surface of aqueous solutions, thereby altering the polarity of solutions and surfaces. The latter property is reminiscent of hydrophobins, which are also small, secreted fungal proteins, but interestingly, the surface-activity-altering properties of cerato-platanins are the opposite of what can be observed for hydrophobins. The so far known biochemical properties of cerato-platanin proteins are summarized in this review, and potential biotechnological applications as well as implications of these properties for the biological functions of cerato-platanin proteins are discussed.

## Introduction

Proteins belonging to the cerato-platanin protein (CPP) family are only found in filamentous fungi, i.e. fungi that produce hyphae as growth structures, or in fungi which have at least a pseudo-hyphal growth stage during their life cycle. The name-giving protein for this family was cerato-platanin (CP) from the plant pathogenic fungus *Ceratocystis platani*, which infects plane trees. CP was first described in 1999 (Pazzagli et al. 1999). Since then, CPPs have been reported from many different filamentous fungi, and it has been recognized that genes encoding CPPs can be found in the genomes of fungi with all kinds of lifestyles, including biotrophic and necrotrophic plant pathogens, human pathogens, mycoparasites, plant-beneficial fungi and saprotrophs (Frischmann et al. 2013; Chen et al. 2013). CPPs are small proteins (12 kDa) that are abundantly secreted into the culture filtrate, but remain also partially bound in the fungal cell wall (Seidl et al. 2006; Boddi et al. 2004; Gonzáléz-Fernandez et al. 2014).

Over the last decade, several studies revealed that CPPs are important players in interactions of fungi with other organisms. Many of the so far reported CPPs are from plant pathogenic fungi, and these proteins are able to act as virulence factors in fungal-plant interactions (Scala et al. 2004; Jeong et al. 2007; Frías et al. 2011). However, in fungi that positively interact with plants, e.g. *Trichoderma* spp., which are used as biocontrol fungi in agricultural applications, they act in a positive way as elicitors of plant defence responses (Djonovic et al. 2006). Further, a member of the CPP family from the human pathogen *Coccidioides immitis* has been described as an antigen (Pan and Cole 1995). Thus, CPPs are readily perceived by other organisms and signal them the presence of a fungus.

With these insights in the effects of CPPs on fungal interactions, several questions came up that still need to be answered: Is the main function of CPPs related to the interaction of fungi with other organisms, or do they have a primary role in fungal growth? Or do they even have multiple roles? In order to answer these questions, a more detailed understanding of the biochemical properties of CPPs is necessary. The studies so far conducted on this topic revealed intriguing properties for this protein family ranging from carbohydrate-binding and carbohydrate-disrupting abilities to protein self-assembly and biofilm formation. Therefore, in this review, the so far known biochemical properties of CPPs, as well as similarities and differences to protein families with related properties, are discussed. Further, we examine what these properties can potentially tell us about the biological functions of CPPs and how these findings could be important for biotechnological applications.

## Features of cerato-platanin proteins: carbohydrate binding and modification

CPPs are small proteins of ca. 12 kDa and contain a signal peptide that targets them to the secretory pathway. They seem to be predominantly secreted and can be readily found in the culture filtrates of fungi (Seidl et al. 2006; Pazzagli et al. 1999; González-Fernández et al. 2014; Frías et al. 2013b). In *C. platani* and *Botrytis cinerea*, CPPs were also already found in the fungal cell wall (Boddi et al. 2004; Frías et al. 2013b).

In the initial studies about CPPs, it was suspected that they are hydrophobin-like proteins (Pazzagli et al. 1999; Seidl et al. 2006), but structural analysis of CP from *C. platani* revealed that there are significant structural differences between CPPs and hydrophobins. CPPs are structurally rather related to expansins, which are proteins associated with carbohydrate-binding and loosening of the cellulose scaffolds in plant cell walls (de Oliveira et al. 2011). However, biochemical analysis of the properties of CPPs showed—somewhat surprisingly—that they have actually both carbohydrate-binding/carbohydrate-loosening properties, similar to expansins (Baccelli et al. 2014), but also the ability to self-assemble and change the polarity of surfaces and solutions, which are properties that are reminiscent of hydrophobins (Frischmann et al. 2013).

The first protein structure that was reported for this protein family was a NMR-derived solution structure for CP. It revealed a globular fold containing two alpha-helices and six beta-strands forming a six-stranded double ψβ-barrel (de Oliveira et al. 2011). The structural fold of CP turned out to be very stable and was preserved over a wide pH range (pH 3 to 9) and up to 76 °C. Further, an *N*-acetylglucosamine-binding pocket forming a shallow groove on one side of the barrel was detected. Another CPP for which structural data are available is SM1 from *Trichoderma virens*. The 3D structure of SM1 has been deposited in the Protein Data Bank (PDB) (accession number 3m3g), but no publication is associated with it yet. Recently, the structures of MpCP1, MpCP2, MpCP3 and MpCP5 from *Moniliophthora perniciosa* were determined (de O Barsottini et al. 2013). All of these CPPs comprise a single domain containing the double ψβ-barrel. This folding is remarkably similar to that found in plant and bacterial expansins, endoglucanases and the plant defence protein barwin (de O Barsottini et al. 2013). Expansins loosen the plant cell wall through a non-enzymatic mechanism (Sampedro and Cosgrove 2005), while endoglucanases catalyse the hydrolysis of cellulose. Barwin proteins belong to the plant pathogenesis-related protein-4 (PR4) family and are generally thought to be involved in plant defence responses (Bai et al. 2013).

For several CPPs (for *Trichoderma atroviride* EPL1, *C. platani* CP, *Ceratocystis populicola* Pop1 and *M. perniciosa* MpCP1-5), it was already shown that they bind to polymeric chitin—which consists of *N*-acetylglucosamine subunits and/or chitin oligomers (Frischmann et al. 2013; Baccelli et al. 2014; de O Barsottini et al. 2013). Interestingly, the chitin-binding site of MpCP5 is in a different region of the protein than in the other MpCPs, suggesting that there is a selective evolutionary pressure on MpCP5 to maintain its carbohydrate-binding properties (de O Barsottini et al. 2013). Chitin is an important structural component of the fungal cell wall, and the peptidoglycan of bacterial cell walls contains also *N*-acetylglucosamine sugars. However, so far, no binding to fungal or bacterial cell walls was detected (Frischmann et al. 2013). Further, none of the so far tested CPPs bind to cellulose, but nonetheless, for CP, expansin-like activity of cellulosic materials was reported (Baccelli et al. 2014). This included weakening of filter paper, fragmentation of crystalline cellulose and breakage of cotton fibres. These effects were also observed for Pop1, albeit weaker than for CP, and it will be interesting to test further CPPs in order to analyse their expansin-like activities in more detail. For MpCP2, it was reported that only an aggregated form, but not soluble protein, was able to fragment cellulose (de O Barsottini et al. 2013). Possibly, there is considerable differentiation among CPPs with respect to cellulose fragmentation. For EPL1, the filter paper assay setup used for CP and Pop1 (Baccelli et al. 2014) was unfortunately not feasible due to excessive foaming of the protein solution (R. Gaderer and V. Seidl-Seiboth, unpublished results).

The biological function of the chitin-binding properties is not clear yet. One possibility is that CPPs are fungal expansins, and their roles might therefore be related to expansin-like activities within the fungal cell wall. Another possibility is that their functions are related to the scavenging of chitin oligomers, e.g. during fungal-plant interactions, which are released due to the action of plant chitinases and would otherwise induce plant defence responses in order to mask the presence of the fungus for the plant, similar to what has been reported for the LysM protein Ecp6 from the plant pathogen *Cladosporium fulvum* (de Jonge et al. 2010). However, since CPPs like EPL1 and its orthologues in several fungi are continuously secreted under many different growth conditions, we suggest that a more general role related to fungal growth, i.e. in the fungal cell wall, is more likely than a specialized function during fungal-plant interactions.

## Features of cerato-platanin proteins: self-assembly and protein biofilm formation

Biochemical analysis of EPL1 from *T. atroviride* revealed that it readily forms protein biofilms at air/water interfaces (Frischmann et al. 2013). A first indication for the self-assembly properties of CPPs from *Trichoderma* were reports on the dimerization of these proteins (Seidl et al. 2006; Vargas et al. 2008). EPL1 is able to form relatively stable dimers, which were still observed after protein-concentration and protein-denaturing steps during 2D gel electrophoresis of a culture filtrate of *T. atroviride* (Seidl et al. 2006). Detailed mass spectrometric analysis of EPL1 showed that the dimer had a double-oxidized tryptophan residue. Subsequently, a comparison of the homologues SM1 from *T. virens* and EPL1 from *T. atroviride* revealed that, although these proteins are overall highly similar (83 % amino acid (aa) identities), *T. virens* has a glycosylation site which is not found in *T. atroviride* (Vargas et al. 2008). This glycan moiety also influences the dimerization tendencies of SM1, which is less prone to form dimers than the non-glycosylated EPL1. This is also of biological relevance because the monomeric form is more efficient in the induction of plant defence responses than the dimer.

**Fig. 1:**
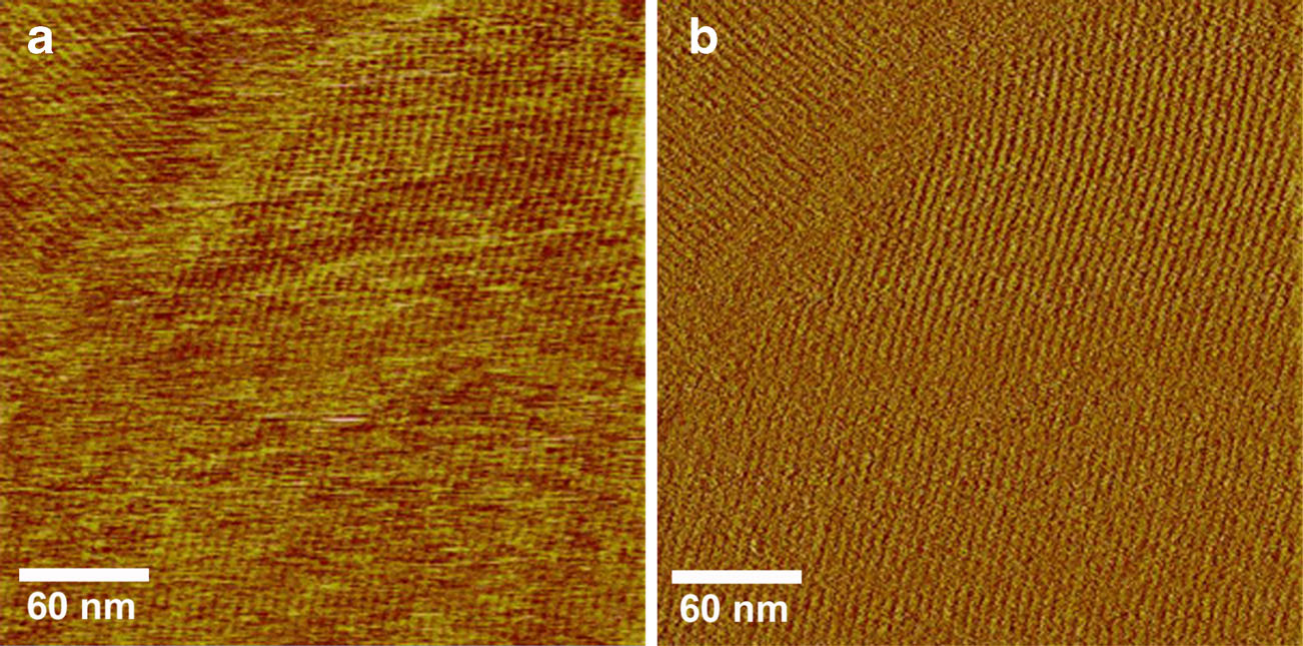
AFM height (**a**) and amplitude error (**b**) images of a highly ordered EPL1 protein layer on a hydrophobic HOPG substrate. The average distance of protein rows is ca. 6 nm. Areas with homogenous protein orientation are in the micrometer range. The selected spot shows an orientation zone boundary and a terrace step of the substrate. Images were taken in tapping mode under PBS buffer, proteins self-assembled in situ. Data scale, 1 nm (**a**) or 10 mV (**b**) from dark to bright

An investigation of the self-assembly potential of EPL1 showed that protein layers can be readily observed under the microscope and are even macroscopically visible after a few minutes of incubation of a protein solution (Frischmann et al. 2013). Self-assembly of EPL1 is reversible, and these protein biofilms can be easily re-dissolved by pipetting or stirring of the solution. High-resolution imaging of EPL1 layers with atomic force microscopy (AFM) revealed that, in these protein layers, EPL1 assembles into rather irregular, meshwork-like structures (Frischmann et al. 2013). However, it should be noted that, by AFM imaging of these protein layers, rather large amounts of protein are deposited on the sample carrier. More recently, we applied in-solution AFM imaging to investigate the self-assembly of EPL1 in more detail, and we were able to show that, on solid/liquid interfaces, it indeed does form highly ordered protein monolayers (Bonazza et al. 2014). Figure 1 shows AFM images of an EPL1 protein layer that was imaged in situ during its formation in buffer. The riffled surface exhibits a periodicity of 6 nm which corresponds to the size of one monomer (Frischmann et al. 2013). In the height image (Fig. 1a), the measurement mode is strictly related to height proportions and reveals the actual 3D topography, but small height differences are difficult to discern in this imaging mode. In the amplitude error image (Fig. 1b), height proportions are more relative to each other, which limits some size measurement options, but circumvents the limitation of displaying just 1-Å-deep riffles beside a several-nanometer-high step in the substrate surface.

Beside the formation of protein biofilms, EPL1 protein solutions have also other surface-activity-altering properties (Frischmann et al. 2013). They exhibit strong foaming, and at higher concentrations, the protein solution shows the tendency to crawl up along needles and pipet tips. Further, it was shown that EPL1 solutions alter the contact angle of aqueous solutions, making them even more hydrophilic, and EPL1 protein layers that are deposited on surfaces can also enhance the polarity effects of surfaces.

For CP and MpCPs, the formation of amyloid-like aggregates was reported although these were observed after prolonged incubation times and harsher incubation conditions (Pazzagli et al. 2009; Sbrana et al. 2007; de O Barsottini et al. 2013). Rapid formation (less than 30 min) of Pop1 aggregates was observed upon contact with hydrophobic surfaces (Teflon beads), and it was shown that in vivo Pop1 is able to interact with the hydrophobic cuticle of leaves (Martellini et al. 2013). The authors therefore proposed that the interaction of CPPs with host plant cuticle waxes could induce their partial unfolding and thereby enhance the recognition of the proteins by the plant.

## Differences and similarities between cerato-platanin proteins and hydrophobins

The ability to self-assemble at interfaces and to alter the polarity of solutions and surfaces is reminiscent of another protein family: hydrophobins. They are a family of small, secreted cysteine-rich proteins that, similar to CPPs, also solely occur in filamentous fungi (Linder et al. 2005; Wösten and Wessels 1997). These proteins self-assemble in the form of an insoluble amphipathic membrane at hydrophobic/hydrophilic interfaces, thereby forming protective surface coatings of fungal structures and aiding in their adherence to surfaces. Hydrophobins are found on the outer surfaces of cell walls of hyphae and conidia, where they mediate interactions between the fungus and the environment. Because the protein surface of hydrophobins contains large hydrophobic and hydrophilic patches, they are able to invert the polarity of surfaces. Biologically, this is for example relevant for fungal hyphae that emerge from an aqueous growth medium to form aerial hyphae and produce spores, which are then covered with a layer of hydrophobins that render them hydrophobic. This facilitates the dispersal of fungal conidia (spores). Due to their unique properties, hydrophobins are also of interest for biotechnological applications, e.g. modification of surface properties and stabilization of foams and emulsions (Linder et al. 2005).

Biochemically, hydrophobins are characterized by the presence of eight positionally conserved cysteine amino acid residues (Linder et al. 2005). Hydrophobins are conventionally grouped into two classes (class I and II) according to their solubility in solvents, hydropathy profiles and spacing between the conserved cysteines. Overall, hydrophobins share only a few conserved residues besides the cysteine patterns. This also indicates that the cysteines are critical for structural reasons, while the other, variable residues give rise to protein variants with specific properties.

The cysteine spacing pattern of class I hydrophobins is CX_(6)_CCX_(9–39)_CX_(5–25)_CX_(5)_CCX_(8–17)_C and of class II hydrophobins, CX(_10)_CCX_(11)_CX_16_CX_(8)_CCX_(10)_C (Seidl-Seiboth et al. 2011; Wösten and Wessels 1997). The disulfide bridges connecting these cysteines span C1–C6, C2–C5, C3–C4, and C7–C8 (Linder et al. 2005). In contrast to that, analysis of the aa sequences of CPPs shows that, in this protein family, the general cysteine spacing pattern is C_(38)_CXXC_(54)_C. For most CPPs, the CXXC motif in this pattern can be confined to C–G–S/T–C. The disulfide bridges that connect the cysteines in CPPs span C1–C2 and C3–C4 (de Oliveira et al. 2011; de O Barsottini et al. 2013). Thus, there are no similarities in the cysteine spacing patterns between hydrophobins and CPPs and also no other sequence similarities. The high numbers of microbial genomes that have been sequenced in the past decade revealed that many fungi have small, secreted, cysteine-rich proteins (Templeton et al. 1994), of which hydrophobins and CPPs are two families that were now already characterized in more detail. However, there are certainly several more protein families among these small, secreted, cysteine-rich proteins that need to be described yet, and it will be interesting to compare the biochemical properties and functions among them.

**Fig. 2:**
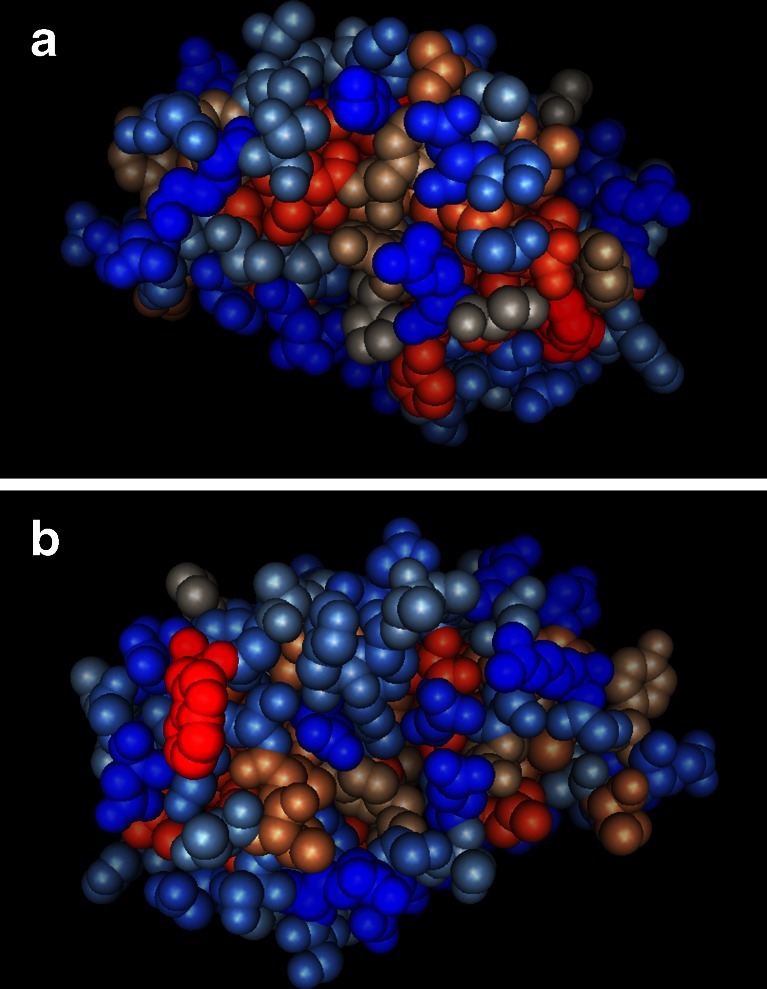
**a**, **b** Modelled 3D structure of EPL1 using SM1 (PDB 3m3g) as template, generated with I-TASSER (Roy et al. 2010). C-score = 1.86. Hydrophobic residues are shown in *shades of red*, hydrophilic residues in *shades of blue*

The surface architecture and the exposure of polar and apolar patches of amino acids are also different between CPPs and hydrophobins. Modelling of EPL1 based on the structure of *T. virens* SM1 (PDB accession number 3m3g) shows that there are some hydrophobic residues on the surface (carbohydrate-binding pockets are also lined with aromatic residues), but there are no large hydrophobic patches on the surface (Fig. 2). On the contrary, the surface seems to be rather hydrophilic, which is in agreement with the good solubility of EPL1 in aqueous solutions.

Another difference between hydrophobins and CPPs is that CPPs are better soluble than hydrophobins. While protein layers of the CPP EPL1 can be easily re-dissolved, the self-assembly of hydrophobin layers is mostly irreversible. Particularly, aggregates of class I hydrophobins are extremely stable and can only be dissolved in strong acids such as trifluoroacetic acid, while class II layers are soluble in aqueous dilutions of organic solvents (Linder et al. 2005).

Beside the differences between CPPs and hydrophobins with respect to their protein sequences and structure, it is important to note that also the surface-altering properties of these two protein families are complementary. EPL1 increases the polarity of surfaces and solutions, which is the opposite of what has been described for hydrophobins, and thus, CPPs could possibly rather be described as ‘hydrophilins’ in this respect. It will be interesting and necessary to determine these parameters for other proteins of the CPP family.

## Gene numbers and gene expression of cerato-platanin proteins

Genome analysis showed that all filamentous fungi belonging to the phyla Ascomycota and Basidiomycota have CP-encoding genes, but their numbers vary strongly among different phyla (Chen et al. 2013). Basidiomycota have often many CP genes, e.g. the nectrotrophic plant pathogen *M. perniciosa*, the causal agent of witches’ broom disease in cacao, has 12 CP genes (de O Barsottini et al. 2013). In contrast to that, fungi belonging to Ascomycota have only between one and three CP-encoding genes (Frischmann et al. 2013; Chen et al. 2013). Further, Ascomycota have usually one strongly conserved homologue of *T. atroviride* EPL1, which was already studied in more detail (see above). The protein sequences of EPL1-homologues are highly similar throughout the Ascomycota, and for several fungi, it was already shown that they are abundantly expressed during different types of growth conditions.

*T. atroviride* has three genes encoding CPPs: *epl1*, *epl2* and *epl3*. Transcriptional analysis of these genes showed that *epl1* is expressed during hyphal growth and mycelial development, while *epl2* expression occurs during sporulation and spore maturation, whereas almost no expression was detected for *epl3* (Frischmann et al. 2013). Similar findings were observed for the corresponding CP-encoding genes from *T. virens*: *sm1*, *sm2* and *sm3* (Gaderer 2013). Therefore, these genes are clearly not co-regulated, and these results indicate that the respective proteins are involved in different stages during fungal growth and development. In *C. platani*, it was reported that the expression levels of *cp* are associated with hyphal growth and the formation of chlamydospores, which are specialized, thick-walled, large spores (Baccelli et al. 2012). However, in *Trichoderma*, no connection between chlamydospore formation and *epl/sm*-gene expression was detected (Gaderer 2013; Frischmann et al. 2013). In other fungi, there is also evidence that CP genes, similar to *epl1*, are expressed during hyphal growth, e.g. in *B. cinerea*, *bcspl1* was found to be expressed under many different growth conditions, whereas no expression was found for *bcspl2*, a second CP gene (Frías et al. 2011). *MgSM1* from *Magnaporthe grisea* was also expressed during different fungal growth stages (Yang et al. 2009). In addition to these gene expression data, the protein EPL1 was found to be the predominant protein in the secretome of submerged *T. atroviride* cultivations with glucose as a carbon source (Seidl et al. 2006).

Despite these indications for a role of CPPs in fungal growth, so far, no specific biological function could be assigned to them. Gene knockout strains did not show any phenotypes related to hyphal growth and development (see below for details) (Frischmann et al. 2013; Djonovic et al. 2007; Frías et al. 2011; Jeong et al. 2007).

In the basidiomycete *M. perniciosa*, gene expression data of the 12 CP genes (*MpCP1-12*) showed complex transcriptional profiles throughout fungal development and specific stages of the pathogenic infestation of the plant, suggesting a specialization of the respective proteins in different biological processes. While the *MpCP1* gene was exclusively expressed during basidiocarp formation, *MpCP2* and *MpCP3* expression occurred in fast-growing mycelium and necrotic-infected seed and fruit, while *MpCP4*, *MpCP5*, *MpCP11* and *MpCP12* were especially found to be expressed during the slow-growing biotrophic phase, when the fungus develops in the apoplast of the plant and the pathogen-host survival battle is established. For *MpCP 6*, *7*, *8*, *9*, and *10*, no gene expression was so far detected (de O Barsottini et al. 2013). With the large numbers of CPPs in some basidiomycetes, it will be interesting to test whether there are also homologues with similar functions or the specialization was rather separate in different fungi.

## (Potential) biological roles of cerato-platanin proteins in fungal growth and dissemination

Despite several efforts to unravel the biological function of CPPs, their roles in fungal development are not clear yet. Their strong expression during hyphal growth and their chitin-binding properties together with their similarity to expansins suggest that they might be involved in fungal cell wall expansion and that this might actually be their primary function (see also the section “Features of cerato-platanin proteins: carbohydrate binding and modification”). However, unfortunately, there is so far still no direct evidence for that, and interestingly, the central question, whether the primary function of CPPs is related to fungal growth and development or rather to the interaction of fungi with other organisms, still remains to be solved. Possibly, this is even different for individual CPPs, as gene expression data might indicate, but as long as we do not understand what—if anything—CPPs do in/on/at the fungal cell wall, we cannot definitely answer this question.

In *T. atroviride* and *T. virens*, *epl1/sm1* and *epl2/sm2* single knockout strains and even *epl1/epl2* double knockout strains were extensively tested for phenotypes related to fungal growth, including germination, hyphal elongation and branching, biomass formation and sporulation (Frischmann et al. 2013; Gaderer 2013; Djonovic et al. 2007). In none of these developmental stages were any differences between the wild-type and the knockout strains detected. Further, growth during different types of stress or the transition of hyphae, e.g. from liquid to solid media or surfaces, was also not different from that of the wild type. Based on the differential expression patterns of *epl1-3* and *sm1-3* in *T. atroviride* and *T. virens*, respectively (see above), it is not likely that the lack of phenotype is due to a compensation effect of other CP-encoding genes.

Also, in other fungi, e.g. *M. grisea*, *B. cinerea* and *Leptoshpaeria maculans*, so far no phenotypes of knockout strains related to fungal growth or sporulation were reported (Frías et al. 2011; Jeong et al. 2007; Wilson et al. 2002).

Another possibility for the functions of CPPs would be that they are connected to chitin utilization, but in *T. atroviride*, no direct inducibility of *epl1-3* genes by chitin was found, suggesting that the proteins EPL1-3 are not involved in aspects related to chitin degradation (Frischmann et al. 2013). Maybe the function of CPPs lies in between fungal growth and fungal interactions?

**Fig. 3:**
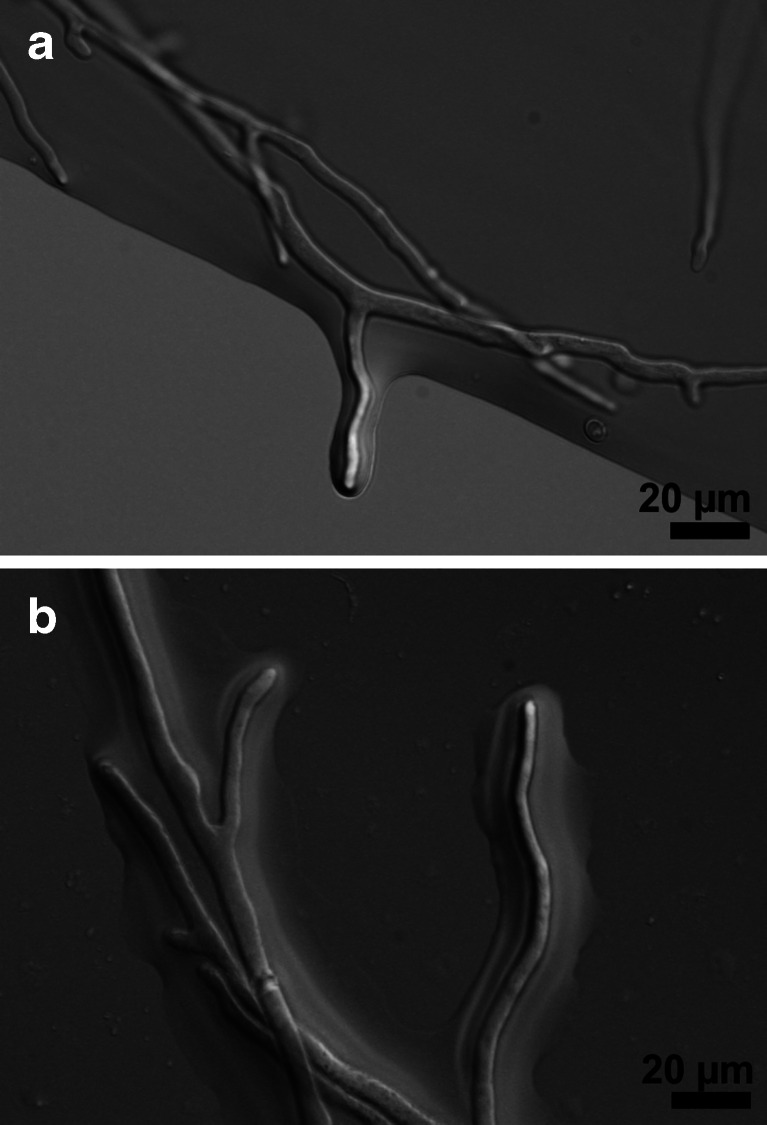
**a**, **b** An aqueous film encloses hyphae of *Trichoderma reesei* that are growing out of a droplet of medium

When *T. atroviride* hyphae grow out of a droplet of water/medium, they are covered with a film of water (Fig. 3). Although no changes in this water film were observed in knockout strains, it could be speculated that the secretion of CPPs influences the adherence of the hyphae to certain environments and with that the interaction of the fungus with its environment. By reducing the surface tension, substrates might become more easily accessible in aqueous environments for the fungus. However, they would not serve their purpose in fulfilling these roles if they would diffuse too far away from the fungus, and maybe due to their chitin-binding properties, they stay partially bound to the cell wall, serving as a reservoir to be partially released under the right growth conditions. It will be difficult to test some aspects of such a hypothesis, particularly since some of these effects might be more significant in the natural environment than under laboratory conditions, but in support of the general idea that CPPs influence the interaction of fungal hyphae with their environment, we recently obtained experimental data that showed that EPL1 is able to alter the surface properties of hydrophobin layers (Bonazza et al. 2014).

## (Potential) biological roles of cerato-platanin proteins in interactions between fungi and other organisms

In fungal-plant interactions, CPPs have been shown to act as virulence factors and elicitors of plant defence responses. For several plant pathogenic fungi, it was reported that CPPs are an important factor for virulence or induce necrosis in plant tissue (Frías et al. 2011, 2013a, b; Scala et al. 2004; Jeong et al. 2007). The eliciting activity of BcSpl1 from *B. cinerea* was mapped to a two-peptide motif on the protein surface (Frías et al. 2013b). A review discussing the plant-related aspects of CPPs in more detail has recently been published (Pazzagli et al. 2014).

Differential timing of defence-related responses induced by CP and Pop1 was found, which the authors suggested could be due to the structural differences between CP and Pop1, i.e. different hydrophobic index and different helix content (Lombardi et al. 2013). In addition to such biological differences between CPPs, which are undoubtedly present, overall, the question of the dose of applied, purified CPPs and/or expression levels in vivo should also be considered when different effects are observed for different CPPs. This might have a profound influence on the observed effects. For example, in *M. grisea*, knockout strains showed reduced virulence, while purified protein applied to wounded leaf tissue showed no phytotoxic effects (Jeong et al. 2007), and ectopic expression in *Arabidopsis thaliana* conferred broad-spectrum disease resistance (Yang et al. 2009).

The elicitation of defence responses by CPPs does not necessarily result in negative consequences such as necrosis, but can also strengthen the plants and confer resistance against microbial pathogens, as was shown for *Trichoderma* spp. *T. virens* SM1 and to a lesser extent *T. atroviride* EPL1 were shown to be an important inducer of plant defence responses (Djonovic et al. 2006; Vargas et al. 2008). Since *Trichoderma* spp. are plant-beneficial biocontrol fungi, the fungal-plant interaction as well as the effect of CPPs is in this case not associated with disease symptoms or necrosis in plants. The induction of defence responses by CPPs can also be conferred artificially by transgenic expression *in planta*. The CP MgSM1 from *M. grisea* was expressed in *A. thaliana*, and subsequently, the plants showed enhanced disease resistance and upregulation of defence-related genes (Yang et al. 2009).

The CPP AgCS (also called CS-Ag, *Coccidioides*-specific antigen) (Cole et al. 1989; Pan and Cole 1995) from the human pathogen *C. immitis*, which causes respiratory mycosis, has been reported to be an antigen. Antibodies of serum from patients with coccidioidomycosis reacted with AgCS that was isolated from the saprobic growth stage of *C. immitis*. In the initial study (Cole et al. 1989), AgCS, purified from culture filtrate, was reported to possess proteolytic activity. In the subsequent study (Pan and Cole 1995), AgCS was overexpressed in *Escherichia coli*, but protease activity of the purified protein was not reported in this follow-up paper. CPPs from other fungi were not tested for protease activity, and it might be something that should be kept in mind although the reported protease activity could have resulted from an impurity of the protein preparation.

In general, it can be concluded with respect to the roles of CPPs in the interactions between fungi and other organisms that CPPs are readily recognized, e.g. by plants or humans, probably due to their abundant secretion by fungi. We suggest that the presence of CPPs signals the other organism reliably the presence of a fungus, but probably dependent on other factors of this interaction, this can lead to different responses with positive or negative consequences for this interaction.

## Possibilities for biotechnological applications of cerato-platanin proteins

Particularly, the surface-activity-altering effects of CPPs are potentially interesting for biotechnological applications, but we are not aware of any ongoing efforts in this respect yet. With native CPPs, stable coating of surfaces is not possible due to their good water solubility, but protein modification, coupling to or mixing with other proteins could enable this. We found, for example, that CPPs and hydrophobins form layers with mixed properties (Bonazza et al. 2014). Further, CPPs could be used to enhance the wettability properties of solutions, which can be of interest for applications where a uniform moistening of a moderately hydrophobic surface is of interest, e.g. spraying of plant protection products or in cleaning agents.

With an increasing understanding of the mode of action of CPPs, their possible use as additives for the induction of plant resistance and defence mechanisms in fertilizers would also be an interesting possibility.

The ability of CP and Pop1 to weaken cellulose—which still needs to be tested for other CPPs—should also be kept in mind for potential applications in cellulose degradation, e.g. bioethanol production from cellulosic plant waste material or paper processing (Himmel et al. 2007).

The strong foaming of the solution of CPP, as has been reported for Epl1 (Frischmann et al. 2013), could also be of interest for the stabilization of foams and emulsions. Further, foaming can be a problem during fermentation of filamentous fungi, which is carried out, for example, in large-scale enzyme production. Therefore, it could be tested whether knockout strains of CPP-encoding genes show favourable behaviour such as less foam formation during fungal fermentations.

Biochemical analysis of more CPPs will be necessary to elucidate the application potential of CPPs further and to reveal which properties are specific for individual CPPs and which properties can be generally found in all members of the CPP family.
